# Preparation and Characterization of Hydrophobin 4-Coated Liposomes for Doxorubicin Delivery to Cancer Cells

**DOI:** 10.3390/ph17111422

**Published:** 2024-10-24

**Authors:** Fatma Hande Osmanagaoglu, Aysegul Ekmekcioglu, Busel Ozcan, Gunseli Bayram Akcapinar, Meltem Muftuoglu

**Affiliations:** 1 Institute of Health Sciences, Department of Medical Biotechnology, Acibadem Mehmet Ali Aydinlar University, 34752 Istanbul, Turkey; handeosmanagaoglu@gmail.com (F.H.O.); aysegul.ekm@gmail.com (A.E.); gunseli.akcapinar@acibadem.edu.tr (G.B.A.); 2 Faculty of Engineering and Natural Sciences, Department of Molecular Biology and Genetics, Acibadem Mehmet Ali Aydinlar University, 34752 Istanbul, Turkey

**Keywords:** Hydrophobin 4, liposome, doxorubicin, drug delivery, nanoparticles

## Abstract

**Background:** The properties of nanoparticle surfaces are crucial in influencing their interaction with biological environments, as well as their stability, biocompatibility, targeting abilities, and cellular uptake. Hydrophobin 4 (HFB4) is a class II HFB protein produced by filamentous fungi that has a natural ability to self-assemble at hydrophobic-hydrophilic interfaces. The biocompatible, non-toxic, biodegradable, and amphipathic properties of HFB4 render it valuable for improving the solubility and bioavailability of hydrophobic drugs. We have investigated the physicochemical properties, cellular uptake, and anticancer effects of empty and Doxorubicin (Dox)-loaded HFB4 liposomes (HFB4L) and compared them to those of PEGylated liposomes (PPL). **Methods:** The *Pichia pastoris* KM71H strain was used for HFB4 purification. Liposomes were prepared through the thin film hydration method and characterized. The cytotoxic effects of free Dox, Dox-HFB4, and Dox-PPL were assessed in MCF7 cells using the SRB Assay. **Results:** All formulations showed good size homogeneity and a spherical shape. The HFB4 coating enhanced the physicochemical stability of Dox-HFB4L over 60 days at 4 °C without significantly affecting Dox release from HFB4L. It increased Dox release at pH 5.4 compared to pH 7.4, indicating higher delivery of drugs into acidic tumor environments, similar to Dox-PPL. While both formulations showed increased cellular uptake compared to free Dox, they exhibited a lower anticancer effect due to the sustained release of Dox. Notably, Dox-HFB4L displayed greater cytotoxicity than Dox-PPL in MCF7 cells. **Conclusions:** HFB4L may offer superior benefits in terms of delivering drugs to an acidic tumor environment in a stable, non-toxic, and sustained manner.

## 1. Introduction

The modification of nanocarrier surfaces with proteins can enhance their stability, biocompatibility, targeting capabilities, and cellular uptake, making it a valuable strategy for designing advanced drug delivery systems [1,2]. The surface properties of nanoparticles can influence their interaction with biological systems, including their circulation time in the bloodstream, their ability to evade the immune system, and their targeting of specific cells or tissues. At the cellular level, it may affect nanoparticle interaction with cells and their ability to be taken up, which is important for drug delivery and therapeutic efficacy. The biocompatibility and increased stability in the biological environment provided by protein-modified nanocarriers contribute to minimizing systemic toxicity and improving pharmacological profiles [3,4,5,6,7,8]. Hydrophobins (HFBs) show promise as surface-modifying proteins for nanoparticles, offering a range of advantages for biomedical and nanotechnology applications [9,10].

Hydrophobin 4 (HFB4) is a class II HFB protein produced by filamentous fungi. HFB4, like other HFBs, is known for its ability to self-assemble into amphipathic layers at hydrophobic-hydrophilic interfaces and to reverse the hydrophilic-hydrophobic character of a surface [11]. This thin and relatively stable monolayer, approximately 10 nm in thickness, provides a convenient protocol for modification to alter the surface physicochemical properties, protein adsorption, biodistribution, and modification [4,12,13]. Their amphipathic nature makes them potentially useful for improving the solubility and bioavailability of hydrophobic drugs. The HFB coating prevents immune recognition of airborne fungal spores, so it plays a role in the prevention of immune response [14]. It has been demonstrated that the human opportunistic fungal pathogen *Aspergillus fumigatus* conidial (spore) surface-class I HFB, RodA, does not activate dendritic cells, alveolar macrophages, and helper T cell immune responses. On the other hand, the lack of a hydrophobin layer on the surface of the spore, either by chemical treatment or genetic change, induces immune activation [14]. In addition, the chemically treated or genetically modified *Fusarium* and *Aspergillus fumigatus* conidia to remove Rod A protein caused increased surface exposure of β1,3-glucan and α-mannose, and cytokine production [15]. Another study with *Aspergillus fumigatus* showed that the surface rodlet protein RodA acts as an inhibitor of phagocytosis, possibly via physicochemical means and by impeding cell wall microbe-associated molecular pattern recognition by macrophage receptors [16]. These results are supported by another cell surface HFB protein, HypA, of *Arthroderma benhamiae*, such that deletion of HypA resulted in “easily wettable” mycelia and conidia, indicating the loss of surface hydrophobicity. Moreover, HypA deletion resulted in increased release of interleukins and tumor necrosis factor-alpha, indicating activation of dendritic cells and human neutrophil granulocytes [17]. In conclusion, the HFB coating on the liposome could confer protection against in vivo immune-system recognition and hence prevent an immune response. Additionally, properties such as biocompatibility, non-toxicity, and biodegradability make HFBs suitable and preferred for drug delivery systems [18,19].

HFBs have been used for either coating the drug directly [10,20,21,22,23,24] or coating the surface of the drug-carrying nanoparticles [4,5]. It was reported that coating with HFBs increased the oral bioavailability of poorly water-soluble drugs by stabilizing drug nanocrystals [20], and controlled and targeted drug delivery by functional surface modification to nanoparticles [21,22]. HFB-1–coated niosomes loaded with doxorubicin (Dox) showed higher encapsulation efficiency, a better-sustained profile, and improved anticancer effects compared to PEG-coated niosomes [5]. Thus, it has been suggested that HFBs may be used as an alternative to PEG to change the surface characteristics of nanocarriers; they may offer several advantages in terms of biocompatibility, stability, and drug delivery efficiency [5]. PEG-coated liposomal Dox was developed to overcome the systemic toxicity of conventional Dox therapy [25]. The development of stealth liposomes by the surface coating of PEG (PEGylation) is used to extend the circulation half-life of liposomes [26]. PEGylation can reduce protein adsorption and thereby confer a stealth effect. On the other hand, it can affect the composition of plasma proteins that bind the carrier and therefore exhibit lower cellular uptake compared to non-covered carriers [5,27]. The PEGylation can also limit cellular interactions with target cells and cellular internalization [28,29,30]. Anti-PEG antibodies present in patients can disrupt the membrane integrity and result in rapid release of encapsulated Dox from the PEGylated liposome, which contradicts the stealth effect of PEGylation [31]. This study aims to evaluate the physicochemical properties and anticancer effects of class II, HFB4-coated empty, and Dox-loaded liposomes and compare them to those of PEGylated liposomes.

## 2. Results and Discussion

### 2.1. Physicochemical Properties of HFBs

The physicochemical properties of HFBs are shown in Table 1. HFB4 was selected for further studies as it has a pH closer to the pH of the human body, which is pH 7.2–7.4. At the physiological pH, HFB4 with a pI of 8.2 will carry a net positive charge, whereas HFBI and HFBII will carry a net negative charge. A positively charged HFB4 at physiological pH will have stronger electrostatic interactions with the negatively charged membranes compared to the negatively charged HFBI and HFBII. Additionally, HFB4 was predicted to have a high aliphatic index, similar to HFBII. Multiple sequence alignment (MSA) results and consensus sequences further show that the core hydrophobin Cysteine motifs and hydrophobicity are well conserved in all three HFBs (Figure S1).

### 2.2. Purification of HFB4 in Pichia pastoris

**Figure 1:**
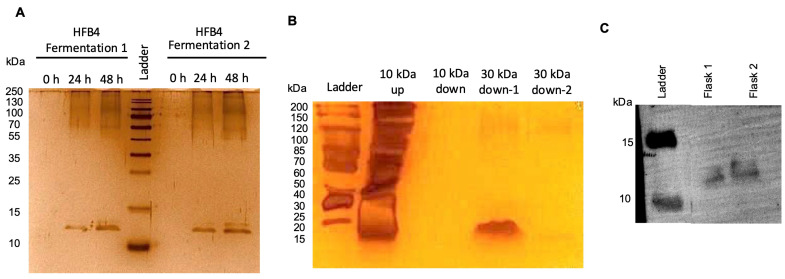
SDS-PAGE of HFB4 production for 48 h in BMM (**A**) Silver stain of purified HFB4 dimer before and after 2-step ultrafiltration process (10 kDa–30 kDa cut-off) (**B**) Western blot analysis of the purified HFB4 dimer (**C**).

*Pichia pastoris* was chosen to purify HFB4 because of its rapid growth rate, allowing for the production of large quantities of protein in a relatively small volume used as an expression host for the production of HFB4 [32,33]. Baffled flasks were used to facilitate higher oxygen rates in production in a 30 °C, 180 rpm incubator shaker for 48 h, and the total protein concentration was 223 µg/mL. After the HFB4 purification, the HFB4 protein concentration was 43 µg/mL. Production of the recombinant HFB4 protein by the *Pichia pastoris* KM71H strain was verified with SDS-PAGE (Figure 1A). The recombinant HFB4 was purified to homogeneity as a dimer using a two-step ultrafiltration process (Figure 1B). The presence of the recombinant HFB4 was further confirmed with Western blot analysis (Figure 1C). In accordance with the previously reported study results [34], HFB4 was found as a dimer of around 13 kDa.

### 2.3. Preparation and Characterization of Empty and Dox-HFB4L and Dox-PPL

Liposomes were prepared in two different DSPE-PEG concentrations as follows: 1. 5%-DSPE-PEG liposomes (5%-PPL) were prepared by mixing cholesterol: L-α-PC: DSPE-PEG in the ratio of 1:0.95:0.05 (mol %); 2. An 8%-PPL was prepared by mixing cholesterol: L-α-PC: DSPE-PEG in the ratio of 1:0.92:0.08 (mol %) [35]. Biswas et al. characterized the 3, 5, and 8 mole % of PPL, and there was not much difference in the average particle size of the liposomes; however, the zeta potential of the liposomes changed significantly upon the addition of (3-carboxypropyl) triphenyl-phosphonium bromide (TPP) [35]. The particle size of 8%-PPL (232.30 ± 2.97) was smaller than that of 5%-PPL (275.01 ± 2.19), and 8%-PPL formulations were used in this study (in the text, 8%-PPL is abbreviated as PPL). HFB4 liposomes (HFB4L) were prepared with the same method by mixing cholesterol: L-α-PC: HFB4 ratio 1:0.92:0.08 (mol %).

The mean particle size, PDI, and zeta potential of empty PPL, Dox-loaded PPL (Dox-PPL), empty HFB4L, and Dox-loaded HFB4L (Dox-HFB4L) are summarized in Table 2. The PDI of all formulations is below 0.2, indicating that the size distributions of all liposomal formulations are relatively monodisperse [36]. The negative zeta potential indicates that the surface charge of all liposomal formulations is negative. Adding sucrose during freeze-drying of Dox-HFB4L and Dox-PPL did not change the particle size, PDI, and zeta potential of the liposomes significantly (*p* > 0.05). The values given in Table 2 show the mean and standard deviation (STD) of three independent experiments.

Two studies used HFBs to produce nanoparticles with various formulations [4,5]. The HFB1 was used to coat the niosome for the delivery of Dox to different cell lines. The particle size of the niosome coated with HFB1 was around 593 nm, and the PDI was over 0.2 [5]. The HFBI-coated curcumin PLGA nanoparticles (HPB PLGA NPs) were obtained by soaking curcumin-loaded PLGA nanoparticles (PLGA NPs) in an aqueous solution of HFBI. The particle size of PLGA NPs (112 nm) increased to 144 nm in HPB PLGA NPs [4]. The particle size of Dox-HFB4L was 253.40 nm (Table 2). One of the properties that influence the stability, bioavailability, efficacy, and safety profile of liposomal drug delivery systems is the particle size. The size of liposomes can affect their distribution, circulation time in the bloodstream, and cellular uptake. Smaller liposomes (typically in the range of 50–250 nm) can penetrate tissues and cells more readily, while larger liposomes may provide a longer circulation time. However, very small sizes can lead to faster clearance by the reticuloendothelial system [37].

**Figure 2:**
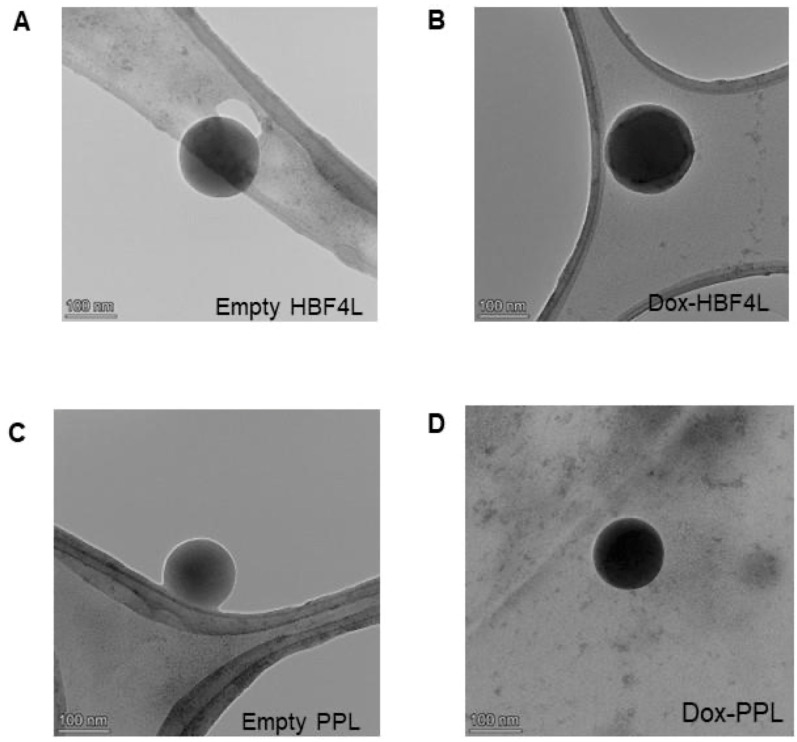
TEM images of HFB4L and PPL. Empty HFB4L (**A**) Dox-HFB4L (**B**) Empty PPL (**C**) Dox-PPL (**D**).

TEM images of empty Dox- HFB4L and Dox-PPL are presented in Figure 2. TEM results show that all liposomal formulations have spherical morphology (Figure 2). Dox-HFB4L and Dox-PPL have darker core parts, indicating Dox loading (Figure 2B,D), while empty HFB4L and PPL have light gray core parts (empty) (Figure 2A,C). Since TEM images were taken under a high vacuum, water in the aqueous core of the liposomes may evaporate, causing the liposomes to shrink as the water evaporates. Therefore, the size of liposomes obtained in TEM images may be smaller than the size determined in DLS [38,39].

### 2.4. Stability of Dox-HFB4L and Dox-PPL

**Figure 3:**
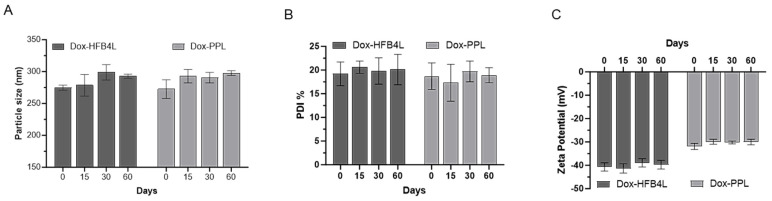
Stability of DoxPPL and Dox-HFB4L for 60 days, at 4 °C. Particle size (**A**), Polydispersity index (PDI) (**B**), zeta potential (**C**). The data are represented as the mean ± STD of three independent experiments.

The encapsulation efficiency of Dox in PPL was found to be 20.9% (equal to 20.9 µM Dox), and that of Dox in HFB4L was found to be 8.2% (equal to 8.2 µM Dox). The encapsulation efficiency of both forms is not high; however, it is sufficient to conduct further analysis in this study. First, it was tested whether Dox-HFB4L was stable at 4 °C without lyophilization by measuring particle size and PDI (Table S1). The particle size of Dox-HFB4L at 4 °C increased 1.6-fold on day 3 compared to that of day 1. The PDI of Dox-HFB4L increased from 0.19 to 0.27, indicating the aggregation of the Dox-HFB4L solutions. Physical treatment with vigorous vortexing resulted in a decrease in size but did not affect the PDI much (Table S1). Vigorous vortexing may also harm the liposome structure and cause drug leakage. Therefore, for the long-term stability of Dox-HFB4L and Dox-PPL, they were lyophilized. Sucrose was added as a lyoprotectant with a sucrose-to-lipid molar ratio of 3:1 before freezing and drying Dox-HFB4L and Dox-PPL [40]. The use of sucrose also facilitated the resolubilization of lyophilized Dox-HFB4L and Dox-PPL stored at 4 °C. To protect against drug leakage, lyophilized Dox-HFB4L and Dox-PPL were resuspended in water by hand shaking for approximately 20 s, and then measurements were taken. The time-dependent changes in particle size (Figure 3A), PDI (Figure 3B), and zeta potential (Figure 3C) of Dox-HFB4L and Dox-PPL at 4 °C were determined (Figure 3). There was no statistically significant change (*p* > 0.05) in the particle size (Figure 3A), PDI (Figure 3B), and zeta potential (Figure 3C) of Dox-HFB4L and Dox-PPL over 60 days, indicating the stability of the formulations.

Since the stability of liposomes is important for effective drug delivery and therapeutic effectiveness, various coating materials, including proteins, have been studied to increase liposome stability [41]. Li et al. have shown that Dox-loaded Transferrin-modified PEG liposomes were stable for 60 days at 4 °C, with a leakage ratio of 0.39% for the Dox [42]. Similarly, a leakage ratio of 0.2% was measured in this study, and the percentage of Dox remained constant during the lyophilization process for 60 days.

Class I (HGFI, H star protein B, and SC3) [20,21,22,23,24] and Class II (HFB1 and HFBII) [4,5,10] HFBs were used for either coating the drug directly or coating the surface of the drug-loaded nanoparticles. In a recent study, the direct coating of curcumin with Class I HGFI showed better stability than Class II HFBI stored in liquid form for 96 h [23]. However, none of these studies investigated the long-term stability of drugs or liposomes coated with HFBs. The instability of liposomes during storage significantly limits their potential as drug delivery systems. The HFB4L and PPL formulations used in this study provided long-term stability for 60 days at 4 °C. Freeze-drying has been suggested as a method to enhance the long-term stability of liposomes [43]. Disaccharides such as sucrose and trehalose are reported to be the most effective lyoprotectants to preserve physical integrity during freeze-drying and rehydration [44]. After 72 h at 4 °C, the liquid forms of Dox-HFB4L started to aggregate. Gentle vortexing to prevent drug leakage was ineffective in dispersing the aggregates, leading to an approximately 2.5-fold increase in particle size compared to day zero. Aggressive vortexing to achieve homogeneous dissolution resulted in drug leakage. Thus, freeze-drying and applying nonreducing disaccharide sucrose with a molar ratio of 3:1 (sugar: lipid) as a lyoprotectant before freeze-drying eliminated these problems and increased stability for 3 months.

### 2.5. In Vitro Dox Release from Dox-HFB4L and Dox-PPL

**Figure 4:**
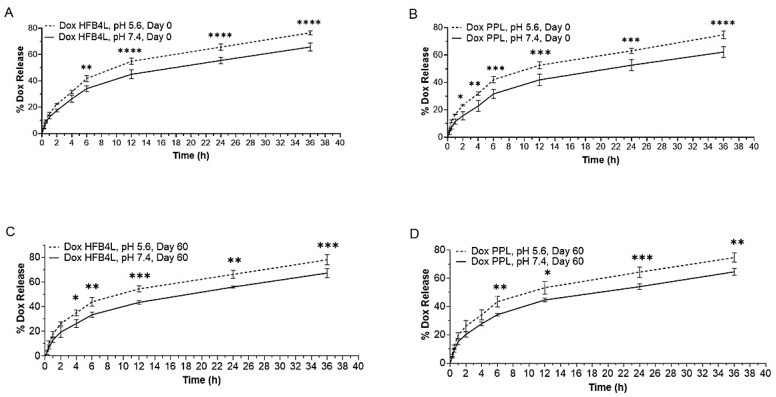
Release profiles of Dox-HFB4L Day 0 (**A**), Dox-PPL Day 0 (**B**), Dox-HFB4L Day 60 (**C**) Dox-PPL Day 60 (**D**) for pH 5.6 and pH 7.4 at different time points. One-way ANOVA using Tukey’s multiple comparison test was conducted for each time point; *p* < 0.05 was accepted as statistically significant. Each experiment was performed in triplicate. * *p* = 0.0103; ** *p* = 0.0035; *** *p* = 0.0007; **** *p* < 0.0001.

The in vitro time-dependent release profiles of Dox-HFB4L and Dox-PPL were analyzed at two different pH levels: pH 7.4 and pH 5.6 at 37 °C, since the pH of tumor tissue is acidic (pH 5.6–6.8) [45,46]. Both Dox-HFB4L and Dox-PPL displayed 30% Dox release in the first 4 h, and their release patterns were similar (Figure 4 and Figure S2). A statistically significant increase in the release of Dox from both Dox-HFB4L and Dox-PPL was observed at pH 5.6 compared to pH 7.4, as illustrated in Figure 4 (*p* < 0.05). The storage of lyophilized Dox-HFB4L and Dox-PPL at 4 °C for 60 days did not significantly affect the release of both liposomes (Figure 4 and Figure S2).

The concentration of Dox released from both Dox-HFB4L and Dox-PPL was statistically significantly higher at acidic pH 5.6 compared to physiological pH 7.4 (*p* < 0.05). This indicates that these formulations may provide an advantage in releasing more drugs in an acidic tumor environment. Class II HFB1 -coated niosomes loaded with Dox released more drugs at acidic pH for 15 h [5]. In this study, the release of Dox from Dox-HFB4L and Dox-PPL remained consistent at various pH levels over a period of two months. Release studies conducted using Class I HFBs did not show any difference in drug release rates at pH 7.4 and pH 5.5 [4,22]. The similarity of release patterns at different pH levels was attributed to the stability of Class I HFBs, such that even at extreme pH, Class I rHGFI maintains its biological activity [22]. In morphological comparison, Class I HFBs are capable of self-assembling into a chemically robust fibrillar structure known as rodlets. However, class II HFBs self-assemble onto a hydrophobic surface, forming an extended protein network that lacks fibrillar morphology [23]. It can be suggested that Class II HFB4 may offer superior benefits over other HFBs for delivering hydrophobic drugs to acidic tumor environments and may be used as an alternative to PEGylated liposomes.

### 2.6. Cellular Uptake and Cytotoxicity of Free Dox, Dox-HFB4L, and Dox-PPL in MCF7 Cells

**Figure 5:**
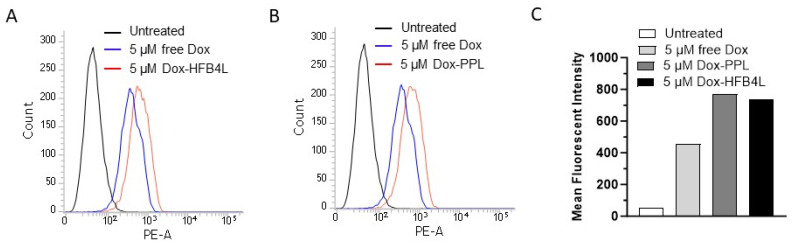
Cellular uptake of free Dox, Dox-HFB4L, and Dox-PPL. Flow cytometry analysis of MCF7 cells after incubation with free Dox (blue), Dox-PPL (red), and untreated cells (black) (**A**). MCF7 cells were incubated with 5 μM free Dox, Dox-HFB4L, and Dox-PPL for 1 h at 37 °C and 5% CO_2_ incubator. The uptake of the liposomes by the cells was quantified using flow cytometry, measuring the signal on the phycoerythrin-area (PE-A) channel. MCF7 cells not incubated with any liposomes were used as a control (**B**). Mean fluorescent intensity (MFI) was obtained from flow cytometry analysis (**C**).

The right shift of the fluorescence intensity (red and blue) in the flow cytometry histogram profile in Figure 5 shows that free Dox, Dox-HFB4L, and Dox-PPL were taken up by MCF7 cells. MCF7 cells exhibited a higher mean fluorescent intensity (MFI) when treated with 5 µM Dox-HFB4L (Figure 5A) and Dox-PPL compared to 5 µM free Dox (Figure 5B). No significant difference in the MFI was observed between Dox-HFB4L and Dox-PPL (Figure 5A–C). Thus, Dox-HFB4L and Dox-PPL have similar and higher cellular uptake profiles compared to free Dox.

**Figure 6:**
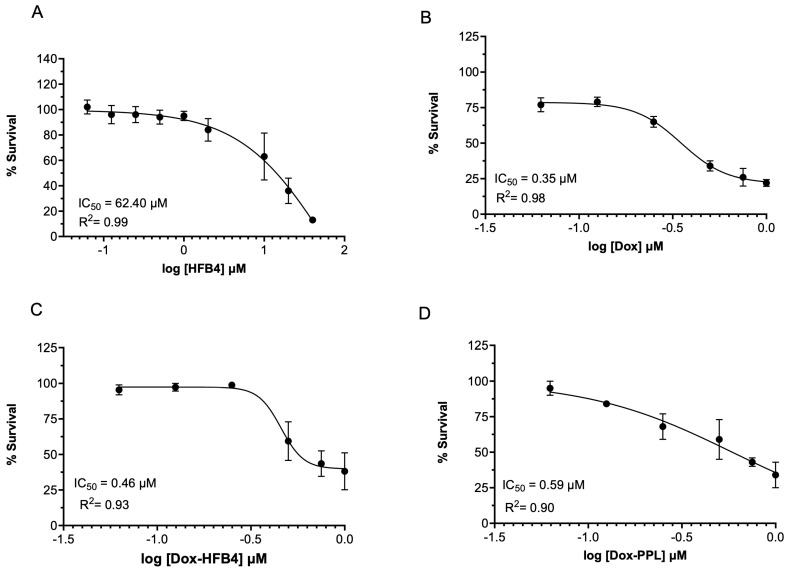
The IC_50_ graphs of HFB4 (**A**), Dox (**B**), Dox-PPL (**C**), Dox-HFB4L (**D**) in MCF7 cells at 72 h.

Initially, we tested the effects of HFB4 on MCF7 cell proliferation to determine the non-toxic concentration of HFB4. The half-maximum inhibitory concentration (IC_50_) of HFB4 was 62.40 µM (R^2^ = 0.99) at 72 h (Figure 6A). We used the non-toxic concentration of 0.4 µM HFB4, which had no impact on MCF7 cell proliferation in HFB4L (Figure 6A). The dose-dependent cytotoxicity and IC_50_ of free Dox, Dox-HFB4L, and Dox-PPL in MCF7 cells are shown in Figure 6. After 72 h, the IC_50_ values of free Dox, Dox-HFB4L, and Dox-PPL were 0.35 μM (Figure 6B), 0.46 μM (Figure 6C), and 0.59 μM (Figure 6D), respectively. The IC_50_ of Dox-HFB4L was 1.28-fold lower than that of Dox-PPL (Figure 6C,D). However, the IC_50_ of Dox-HFB4L was 1.32-fold higher than that of free Dox (Figure 6B,C), and the IC_50_ of Dox-PPL was 1.67-fold higher than that of free Dox (Figure 6B,D).

Although both Dox-HFB4L and Dox-PPL displayed enhanced cellular uptake in comparison to free Dox, they demonstrated a reduced anticancer effect as a result of the sustained release of Dox. Consistent with this, the anticancer effect of the formulation was reduced when niosomes were coated with HFB-1 or PEG [5]. Notably, Dox-HFB4L exhibited higher cytotoxicity than Dox-PPL in MCF7 cells. Transferrin targeting the transferrin receptors, which are highly expressed on the surfaces of tumor cells, was used to modify PEG liposomes [42]. Similar to this study, transferrin-modified PEG liposomes increased the cellular uptake of entrapped Dox into HepG2 cells compared to Dox-loaded PEG liposomes. However, Dox-loaded Transferrin-modified PEG liposomes were more cytotoxic to HepG2 cells at 48 h compared to Dox-loaded PEG liposomes [42].

## 3. Materials and Methods

### 3.1. Materials

*E. coli* DH5α and *P. pastoris* KM71H strains were from Thermo Fisher, (Billings, MT, USA). L-α-Phosphatidylcholine (L-α-PC), cholesterol, phosphate-buffered saline (PBS), biotin, and lithium acetate were from Sigma-Aldrich (St. Louis, MO, USA). 1,2-distearoyl-sn-glycero-3-phosphoethanolamine-N-[methoxy(polyethyleneglycol)-2000] (ammonium salt) (18:0 PEG2000 PE, DSPE-PEG) was from AvantiPolar lipids (Alabaster, AL, USA). Dichloromethane (DCM), sodium sulfate, sodium acetate trihydrate, acetic acid (glacial), ammonium sulfate, potassium dihydrogen phosphate, and dipotassium hydrogen phosphate were from Merck (Taufkirchen, Germany). Dulbecco’s phosphate-buffered saline (DPBS), Penicillin/Streptomycin (Pen/Strep), Zeocin, SYBR SafeDNA Gel Stain, 6x-His-Tag Monoclonal Antibody (3D5), AP, SACI, 1-Step™ NBT/BCIP, Pierce BCA Protein Assay Kit, and Pierce Silver Stain Kit were from Thermo Scientific (Waltham, MA, USA). The cellulose dialysis membrane (10 kDa) was from CelluSep (Miami, FL, USA). Uranyl acetate was from Electron Microscopy Sciences (Hatfield, PA, USA). Dox was from Santa Cruz Biotechnology (Hatfield, PA, USA). Fetal bovine serum (FBS) and Dulbecco’s modified eagle medium (DMEM) were from Gibco (Billings, MT, USA). The SRB Assay kit was from Abcam (Cambridge, UK). The MCF7 cell line was from the American Type Culture Collection (Manassas, VA, USA). Tween-80, Laura-Bertani Medium, Yeast Extract Peptone Dextrose Medium (YPD), and Yeast Nitrogen Base (YNB) without amino acids and ammonium sulfate were from Research Products International (Mt Prospect, IL, USA). The Qiaprep Spin Miniprep Kit was from Qiagen (Germantown, MD, USA). Glycerol and methanol were from Isolab (Eschau, Germany). The Genomic DNA Clean and Concentrator 25 was from Zymo Research (Irvine, CA, USA).

### 3.2. Bioinformatics Analysis

Core HFB regions of HFB1 and HFB2 from *Trichoderma reesei* and HFB4 from *Trichoderma virens* were used in all analyses. Protein physicochemical properties were calculated based on the protein sequences using the EXPASY ProtParam web tool (https://web.expasy.org/protparam/; accessed on 15 September 2021) [47]. Multiple sequence alignment of the core regions of the HFBs (HFBI, HFBII, and HFB4) was performed using Clustal ω [48]. The consensus sequences were calculated using MView [48].

### 3.3. Production and Purification of HFB4

The expression, production, and purification of HFB4 were performed in the *Pichia pastoris* KM71H strain as described in the Invitrogen EasySelect ™ Pichia Expression Kit user manual. Briefly, the synthetic and codon-optimized *Hfb4* gene, encoding the 65 amino acid core region of HFB4 (Uniprot ID: A0A7D5LY27), was cloned between the Eco-RI-*XbaI* sites of the pPICZαA vector. The vector was transformed into competent *E.coli* DH5α cells using heat shock. Transformants were grown on low salt LB agar plates containing 25 µg/mL Zeocin. Positive colonies were verified with colony PCR [49]. The recombinant plasmid with the synthetic *hfb4* was purified using a Qiagen plasmid kit according to the manufacturer’s protocol. The plasmid was linearized with *SacI* and transferred to a competent *Pichia pastoris* KM71H strain through electroporation [50]. The transformants were selected using YPD agar plates containing 100 µg/mL Zeocin, and a second verification was performed using colony PCR with AOX primers. Selected colonies were first grown in Buffered Minimal Glycerol Medium and induced at 28 °C and 200 RPM for 48 h in minimal methanol medium containing 0.5% (*v*/*v*) methanol for recombinant production. One ml samples were collected every 24 h, and 0.5% (*v*/*v*) methanol was added to the medium. The supernatant was collected through centrifugation at the end of the submerged fermentation and stored at +4 °C. The supernatant was concentrated using a 10 kDa and 30 kDa cut-off ultrafiltration cassette (Sartorius, Vivaspin) to remove higher molecular weight *Pichia* proteins. HFB4 protein, found as a dimer at 13 kDa, was purified. Protein concentrations were measured using the BCA assay. The purified HFB4 protein was separated on 15% SDS-polyacrylamide gels using the PageRuler Prestained Protein Ladder and transferred to a polyvinylidene fluoride membrane. The membrane was blocked in 5% non-fat dry milk with TBST (10 mM Tris–HCl, pH 8.0, 150 mM NaCl, 0.1% Tween-20) followed by exposure to alkaline phosphatase-conjugated mouse 6x-His Tag monoclonal antibody (AP primer antibody) (1:1000). the membrane was then exposed to the One-step NBT/BCIP substrate according to the manufacturer’s protocol and visualized using the ChemiDoc MP Imaging System (Bio-Rad Laboratories, Hercules, CA, USA). Silver staining was performed using the Pierce Silver Stain Kit according to the manufacturer’s protocol.

### 3.4. Preparation of Empty and Dox-Loaded Liposomes Coated with HFB4 or DSPE-PEG

Each liposome was prepared using Bangham’s thin film hydration method, as described previously, with some modifications [1,24]. Empty DSPE-PEG liposomes (PPL) were prepared by mixing cholesterol, L-α-PC, and DSPE-PEG in the ratio of 1:0.92:0.08 (mole %). Briefly, all ingredients were dissolved in dichloromethane (DCM), and then a lipid film was formed in a rotary evaporator. The excess amount of DCM was removed using a vacuum desiccator. The dried lipid film was hydrated with DPBS (pH 7.4) and allowed to self-assemble overnight at 4 °C. HFB4 liposomes (HFB4L) were prepared using the same method explained above with the following modifications. First, cholesterol and L-α-PC were mixed in a ratio of 1:0.92 (mole %) in DCM, and the thin film hydration method was applied. After the hydration of the dried lipid film with DPBS (pH 7.4), HFB4 was added to the solution (final cholesterol: L-α-PC: HFB4 ratio 1:0.92:0.08) and homogenized with a magnetic stirrer at room temperature. Both PPL and HFB4L were sonicated for 30 min with an ultrasonic frequency of 35 kHz at room temperature while ice molds were utilized to maintain a consistent temperature in the ultrasonic bath (Bandelin Sonorex GT 1003 M-C). the suspension was then centrifuged at high speed at 200,000× *g*, 4 °C for 16 h to eliminate any excess ingredients. The supernatant was discarded, and the pellet was washed twice. The final liposomal formulation was obtained by filtering through a 0.45 µm filter, followed by a 0.22 µm filter. The same procedure was applied for Dox-loaded HFB4L (Dox-HFB4L) and Dox-loaded PPL (Dox-PPL) by adding 100 µM Dox to the lipid mixture in DCM before the thin film formation step. All liposomal formulations were freeze-dried and stored in lyophilized form to achieve long-term stability. Various studies have shown that adding sucrose as a lyoprotectant during the freeze-drying of the liposome improves the physicochemical stability of the liposome [44,51,52,53]. Sucrose was used as a lyoprotectant before freeze-drying with a sucrose-to-lipid molar ratio of 3:1. Freeze-drying was conducted at −70 °C at a pressure of 0.1 mbar for 48 h (Labconco, Freezone 6 Plus). The freeze-dried samples were stored at 4 °C.

### 3.5. Characterization of Liposomes

The stability of the liposomes was evaluated by dissolving the lyophilized empty PPL, Dox-PPL, empty HFB4L, and Dox-HFB4L in distilled water, shaken manually for 20 s, and then measuring their particle size, particle size distribution (PDI), and zeta potential (surface charge) using the dynamic light scattering (DLS) technique (LiteSizer 500; Anton Paar, Graz, Austria). The particle size, PDI, and zeta potential of freshly prepared Dox-PPL and Dox-HFB4L without sucrose were measured using the DLS technique. DLS and PDI measurements were made without dilution using the Anton Paar LiteSizer 500 (Graz, Austria) with a disposable cuvette (Sarstedt Cuvette Polystyrene 10 × 10 × 45 mm). Zeta potential measurements were performed by mixing 50 µL of the liposome sample with 450 µL of 1 mM NaCl using the Anton Paar LiteSizer 500 with an Omega cuvette (Anton Paar). The parameters were set to the Smoluchowski approximation at a target temperature of 25 °C, automatic 200 V power adjustment, and 20 runs at manual quality, with water as the solvent. The average particle size was represented as mean ± standard deviation (STD) of three independent experiments. The characterization of liposome morphology was conducted using a transmission electron microscope (TEM, Talos L120C, Billings, MT, USA). Ten µL of liposome were placed onto a copper grid (LC300-Cu-25 Lacey/Carbon 300 Mesh), and then 2% uranyl acetate was applied to the sample on the grid for negative staining.

### 3.6. Encapsulation Efficiency

The amount of the encapsulated Dox in Dox-PPL and Dox-HFB4L was measured using a spectral scanning multimode reader (VarioSkan Flash, Thermo Scientific, Billings, MT, USA). Initially, a calibration curve for Dox (serial dilutions from 2 µM to 20 µM in 1XPBS) was created using excitation/emission spectra at 470/490–800 nm, the wavelength at which drug molecules exhibit maximum fluorescence intensity at 595 nm (λmax). The linear line Equation (1) from the calibration curve was used to calculate Dox concentration.

(1) y=mx+n

where,

y = Relative Fluorescence Unit (RFU);

x = concentration in µM.

To determine the Dox concentration of Dox-PPL and Dox-HFB4L, the liposome samples were mixed with ethanol in a 1:1 ratio and vortexed to burst and allow them to release their Dox content. Then, the fluorescence intensity of the solution was measured at the spectrum of excitation/emission 470/490–800 nm. The RFU value, which gives the maximum fluorescence intensity at 595 nm (λmax), was used to determine unknown Dox concentrations according to Equation (2).

(2) $$y\left(RFU\right)=3.24\left(conc\right)x+9.35(R^{2}=0.98)$$

Encapsulation efficiency was calculated by comparing the final Dox concentration of liposome calculated above to the starting Dox concentration of 100 µM. The percentage of encapsulation efficiency was calculated according to Equation (3) [54].

(3) $$Encapsulation\;effeciency\;\%=\frac{Dox\;Concentration\;of\;liposome}{Dox\;concentration\;in\;liposome\;preperation\;step}\times 100$$

### 3.7. In Vitro Drug Release

The release of Dox from the liposome formulations was evaluated at different pH levels. A closed dialysis bag (10 kDa) containing 500 μL of both Dox-loaded HFB4L and Dox-loaded PPL at a final concentration of 8.2 µM was placed in a 50 mL falcon tube containing 40 mL of citrate buffer (pH 5.6) or PBS buffer (pH 7.4). For each liposome’s pH level, a triplicate experiment setup was prepared (*n* = 3). The buffer was stirred at 100 rpm at 37 °C, and 200 µL samples were collected at 15 min, 30 min, 45 min, 1 h, 2 h, 6 h, 12 h, 24 h, and 36 h. A fresh buffer was added to keep the release medium volume constant. Dox content released into the buffer was determined by fluorometric measurement at 595 nm without diluting the sample using a multimode reader (VarioSkan Flash). Since Dox released from the dialysis bag to the buffer was eighty times diluted, a new calibration curve for Dox (serial dilutions from 0.1 µM to 0.8 µM and 0.02 µM to 0.4 µM) was created at 595 nm. The concentration and corresponding total weight in the release media were calculated according to the calibration curve Equation (4) for pH 7.4, and Equation (5) for pH 6.5 as follows:

(4) $$y=6.50x+0.02\;(R^{2}=0.99)$$

(5) $$y=5.31x+0.02\;(R^{2}=0.99)$$

where,

y = Relative Fluorescence Unit (RFU);

x =concentration in µM.

The released amount of Dox in the buffer was presented as the cumulative release in weight and percentual release was calculated by comparing cumulative Dox released by weight vs. initial Dox weight in the dialysis bag.

### 3.8. Cellular Uptake of Liposomes

MCF7 cells were cultured in DMEM supplemented with 10% FBS and 1% Pen/Strep and maintained in a 37 °C, 5% CO_2_ incubator. To analyze the cellular uptake of liposomes, 300,000 cells/well were seeded in 6-well plates in complete DMEM without phenol red. The next day, the cells were incubated with 5 µM Dox, Dox-PPL, and Dox-HFB4L for 1 h in serum-free DMEM without phenol red at 37 °C. Then the cells were washed twice with 1× DPBS and collected using trypsin. Pellets were obtained by centrifugation at 600× *g* for 5 min, then washed with ice-cold 1xDPBS twice and resuspended in 400 µL of 1xDPBS. Tubes were read using a BD FACSVerse flow cytometer, equipped with a 550 nm laser. The cells were gated using forward vs. side scatter to exclude debris and dead cells before being analyzed by FACS with 10,000 cell counts. The data were analyzed using the PE-A channel with BD FACSVerse software (version 4.9.0.0).

### 3.9. In Vitro Cytotoxicity of Empty and Dox-Loaded Liposomes

The cytotoxicity of free and liposomal formulations of Dox was determined in MCF7 cells using the SRB Assay kit (Abcam) according to the manufacturer’s instructions. Briefly, MCF7 cells were seeded on a 96-well plate (5000 cells/well) in complete growth medium and incubated overnight. Serial dilutions of Dox and liposomal Dox formulations were applied to the wells, and the plate was incubated for 72 h at 37 °C in a 5% CO_2_ incubator. the SRB solution was then added to each well, followed by the SRB kit’s protocol. The absorbance of each well was measured at 565 nm using a plate reader (Powerwave XS2, BioTek, Winooski, VT, USA). All experiments were performed in triplicates as three independent experiments. The IC_50_ values were calculated using GraphPad Prism 9.0.2 software.

### 3.10. Statistical Analysis

All data are presented as the mean ± STD of three experiments. Statistical analysis was performed using one-way ANOVA with multiple comparisons by Tukey post-hoc tests for multiple comparisons and the GraphPad Prism 9.2.0 software. *p* < 0.05 was considered statistically significant.

## 4. Conclusions

This study investigated the physicochemical properties and anticancer effects of class II HFB4-coated empty and Dox-loaded liposomes, comparing them to those of PEGylated liposomes. The uncoated liposomal formulations were not included due to their many disadvantages over the stealth liposomes. The uncoated liposomes are less stable, have shorter circulation times, and off-target effects, exhibit higher rates of leakage, and have shorter shelf lives compared to HFB4-coated liposomes. The HFB4 coating increased the physicochemical stability of Dox-HFB4L for 60 days at 4 °C without significantly affecting Dox release from HFB4L and increased Dox release at acidic pH compared to physiological pH. Similar characteristics were observed in Dox-loaded PEGylated liposomes. While both formulations showed increased cellular uptake compared to free Dox, they exhibited a lower anticancer effect due to the sustained release of Dox. Notably, Dox-HFB4L displayed greater cytotoxicity than Dox-PPL in MCF7 cells. In conclusion, this study demonstrated that coating liposomes with HFB4 offers superior benefits in terms of stable, non-toxic, and sustained delivery of hydrophobic drugs to the acidic tumor environment; however, further research is required to improve the encapsulation efficiency and investigate the in vivo efficacy of the formulations.

## Figures and Tables

**Table 1:** Physiochemical properties of HFBI, HFBII, and HFB4. Expasy ProtParam Tool (https://web.expasy.org/protparam (accessed on 15 September 2021) is used to predict the properties of HFBs.

	Molecular Weight (kDa)	Theoretical pI	Total Number of Positively Charged Residues (Arg + Lys)	Instability Index	Aliphatic Index	Grand Average of Hydropathicity (GRAVY)
HFBI	6.6	6.0	3.0	29.7	89.8	0.4
HFBII	6.5	5.4	3.0	−1.3	102.2	0.8
HFB4	6.4	8.2	4.0	14.2	97.4	0.5

**Table 2:** The average particle size, particle size distribution (PDI), and zeta potential of lyophilized empty PPL, Dox-PPL, emptyHFB4L, Dox-HFB4L, and freshly prepared Dox-PPL (-Sucrose) and Dox-HFB4L (-Sucrose).

	Particle Size (nm)	PDI	Zeta Potential (mV)
Empty PPL	232.30 ± 2.97	0.20 ± 0.04	−9.91 ± 0.70
Dox-PPL	260.15 ± 4.63	0.19 ± 0.03	−36.13 ± 3.71
Empty HFB4L	233.10 ± 3.29	0.20 ± 0.01	−40.93 ± 0.33
Dox-HFB4L	253.40 ± 8.33	0.19 ± 0.02	−41.63 ± 1.58
Dox-PPL (-Sucrose)	252.60 ± 0.31	0.16 ± 0.07	−34.85 ± 577
Dox-HFB4L (-Sucrose)	252.95 ± 14.11	0.18 ± 0.05	−42.73 ± 2.14

## Data Availability

Data is contained within the article.

## Supplementary Materials

The following supporting information can be downloaded at: https://www.mdpi.com/article/10.3390/ph17111422/s1, Figure S1: Multiple Sequence Alignment of HFBI, HFBII, and HFB4 with Clustal Omega. (A). Figure S2. Release profiles of Dox-HFB4L Day 15 (A), Dox-PPL Day 15 (B), Dox-HFB4L Day 30 (C), Dox-PPL Day 30 for pH 5.6 and pH 7.4 at different time points. Table S1. The average particle size, particle size distribution (PDI), and zeta potential of Dox-HFB4L without lyophilization.
