# Supplementary material for: Preparation and Characterization of Hydrophobin 4-Coated Liposomes for Doxorubicin Delivery to Cancer Cells
Source: Pharmaceuticals (Basel). 2024 Oct 24;17(11):1422. doi: 10.3390/ph17111422 (PMC11597365; doi:10.3390/ph17111422)
Supplement: Supplementary file 1 [file pharmaceuticals-17-01422-s001.zip › pharmaceuticals-3252682-supplementary.pdf]

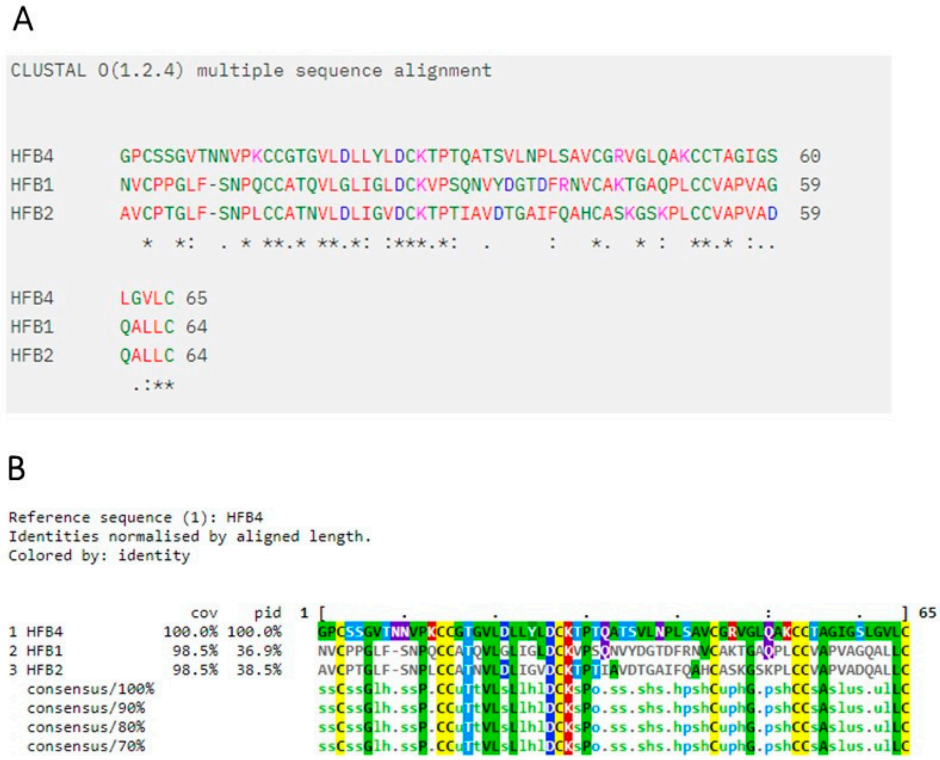

Figure S1. Multiple Sequence Alignment of HFBI, HFBII and HFB4 with Clustal Omega. (A) Mview visualisation of MSA exhibiting conserved residues (B).

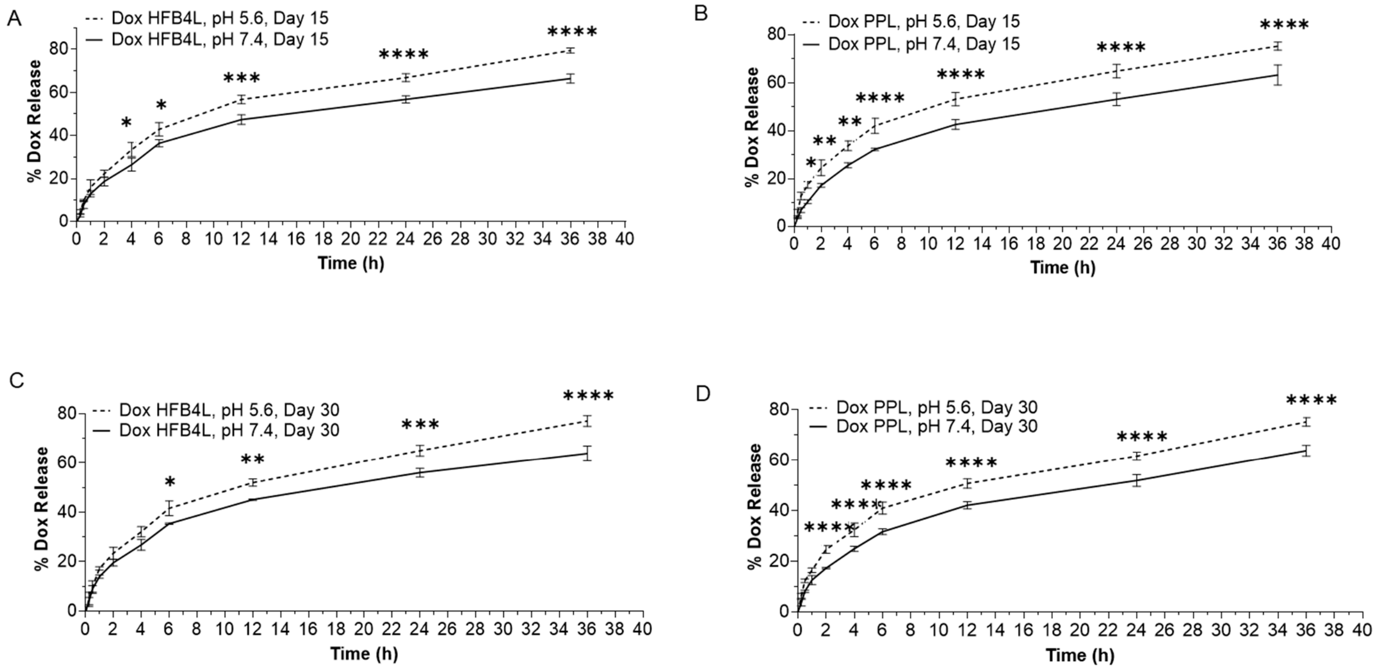

Figure S2. Release profiles of Dox-HFB4L Day 15 (A), Dox-PPL Day 15 (B), Dox-HFB4L Day 30 (C), Dox-PPL Day 30 for pH 5.6 and pH 7.4 at different time points. One-way ANOVA using Tukey's multiple comparison tests was conducted for each time point; $p < 0.05$ was accepted as statistically significant. Each experiment was performed in triplicate. *p= 0.0103; **p= 0.0035; ***p= 0.0007; ****p= < 0.0001.

Table S1. The average particle size, particle size distribution (PDI), and zeta potential of Dox-HFB4L without lyophilization.

	**Day 0 at 4 ºC**		**Day 3 at 4 ºC**	
	**Particle size (nm)**	**PDI**	**Particle size (nm)**	**PDI**
Dox-HFB4L	213.90 ± 2.12	0.19 ± 0.06	341.23 ± 13.55	0.27 ± 0.03
Dox-HFB4L (1 min vortex)	-	-	316.60 ± 19.54	0.26 ± 0.01
Dox-HFB4L (2 min vortex)	-	-	249.73 ± 5.06	0.24 ± 0.01
